# The influence of particle concentration and bulk characteristics on polarized oceanographic lidar measurements

**DOI:** 10.1002/lno.12088

**Published:** 2022-04-29

**Authors:** Brian L. Collister, Richard C. Zimmerman, Charles I. Sukenik, William M. Balch, Victoria J. Hill

**Affiliations:** ^1^ Department of Ocean and Earth Sciences Old Dominion University Norfolk Virginia USA; ^2^ Department of Physics Old Dominion University Norfolk Virginia USA; ^3^ Bigelow Laboratory for Ocean Sciences East Boothbay Maine USA

## Abstract

Oceanographic lidar measurements of the linear depolarization ratio, *δ*, contain information on the bulk characteristics of marine particles that could improve our ability to study ocean biogeochemistry. However, a scarcity of information on the polarized light‐scattering properties of marine particles and the lack of a framework for separating single and multiple scattering effects on *δ* have hindered the development of polarization‐based retrievals of bulk particle properties. To address these knowledge gaps, we made single scattering measurements of *δ* for several compositionally and morphologically distinct marine particle assemblages. We then used a bio‐optical model to explore the influence of multiple scattering and particle characteristics on lidar measurements of *δ* made during an expedition to sample a mesoscale coccolithophore bloom. Laboratory measurements of linear depolarization revealed a complex dependency on particle shape, size, and composition that were consistent with scattering simulations for idealized nonspherical particles. Model results suggested that the variability in *δ* measured during the field expedition was driven predominantly by shifts in particle concentration rather than their bulk characteristics. However, model estimates of *δ* improved when calcite particles were represented by a distinct particle class, highlighting the influence of bulk particle properties on *δ*. To advance polarized lidar retrievals of bulk particle properties and to constrain the uncertainty in satellite lidar retrievals of particulate backscattering, these results point to the need for future efforts to characterize the variability of particulate depolarization in the ocean and to quantify the sensitivity of operational ocean lidar systems to multiple scattering.

Marine ecosystems are dynamic in space and time, requiring measurements across a broad range of spatiotemporal scales to constrain their variability. For the past four decades, ocean color remote sensing satellites have provided the primary means for measuring phytoplankton biomass at synoptic scales across the surface ocean (Jamet et al. 2019). However, these techniques are limited in scope by their reliance on the sun as a passive radiation source. Ocean color measurements represent a daytime, surface‐weighted average over the ocean's first optical depth (Gordon and McCluney 1975), missing deep phytoplankton populations and providing no information on their vertical structure. This “missing” vertical information introduces systematic error into primary production estimates, as the vertical distribution of biomass plays a key role in determining its exposure to factors that control growth and loss (Hill and Zimmerman 2010; Schulien et al. 2017). Oceanographic lidar is the only remote sensing technique that offers to fill this observational gap by providing a means to measure the vertical distribution of marine ecosystems remotely via the range‐resolved detection of a backscattered laser pulse (Hostetler et al. 2018).

In addition to revealing the vertical structure of particle concentration in the upper ocean, polarimetric lidar can provide information on the bulk properties (e.g., shape, size, and compositional characteristics) of a particle assemblage. The most common application of polarimetric lidar involves the emission of linearly polarized light and detection of the parallel and orthogonal polarization components of the backscattered return. When multiple scattering is a negligible component of the return signal, the linear depolarization ratio, *δ* (i.e., the ratio of the cross‐ to co‐polarized returns), can be defined in terms of the second diagonal element of the normalized scattering matrix at a scattering angle of *π* radians, **M**_22_(*θ* = *π*):

(1) $$\delta =\frac{1-M_{22}\left(\pi \right)}{1+M_{22}\left(\pi \right)}$$

**M**_22_(*π*) is an inherent optical property that describes the propensity for a scattering event to depolarize light that is initially linearly polarized, and it exhibits dependencies on the distribution of shape, size, and composition within a particle assemblage. If the single scattering condition is satisfied, **M**_22_(*π*) can be estimated from *δ* by rearranging Eq. 1:

(2) $$M_{22}\left(\pi \right)=\frac{1-\delta }{1+\delta }$$

Relationships between **M**_22_(*π*) and bulk particle properties thus provide a framework for retrieving particle characteristics that are relevant to their functional role in biogeochemical ocean processes from lidar measurements of *δ*. Multiple scattering also increases *δ* with distance at a rate that depends on the scattering coefficient, *b*, the shape of **M**_22_(*θ*) in the forward direction, and the lidar field of view (Zege and Chaikovskaya 1999; Vasilkov et al. 2001), thus convolving the effects of particle concentration and bulk particle characteristics on the value of *δ*. For this reason, it is important to distinguish between **M**_22_(*π*), an inherent optical property, and *δ*, a lidar measured parameter that is sensitive to **M**_22_(*θ*), multiple scattering, and instrument geometry.

Many successful applications of polarimetric lidar have come from the atmospheric lidar community, where profiles of *δ* have been used to measure the thermodynamic phase and orientation of cloud particles (Noel and Sassen 2005; Hu 2007), classify aerosol types (Burton et al. 2012), and characterize cloud droplet size distributions (Roy et al. 1999). Early polarization techniques relied on the absence of linear depolarization by homogeneous spherical particles in the exact backscattering direction (i.e., **M**_22_(*π*) = 1) to separate scattering returns from spherical and nonspherical particles (Sassen 2005). Lidar radiative transfer models were used to explore the influence of multiple scattering on *δ* and to develop techniques for retrieving information on particle size and concentration contained in the multiple scattering component of the return (Platt 1981; Hutt et al. 1994). Advances in light‐scattering simulations aided in the development of advanced polarization lidar algorithms for distinguishing between nonspherical particles of varying size, shape, and composition (David et al. 2013; Mehri et al. 2018).

Churnside (2008) was the first to suggest that polarimetric lidar could be used to derive the bulk properties of aquatic particles after showing that lidar measurements of *δ* exhibited patterns that were spatially consistent with expected shifts in particle composition and morphology between coastal and offshore waters. Subsequent studies developed empirical relationships between *δ* and the bulk properties of marine particle assemblages, bolstering the idea that *δ* could be used to retrieve information on marine particle characteristics (Collister et al. 2018; Dionisi et al. 2020; Schulien et al. 2020). However, these empirical field studies offered limited mechanistic insight into the sensitivities of **M**_22_(*π*) to particle shape, size, and composition. Variability in **M**_22_(*π*) was not apparently dominated by any single particle property across multiple investigations, with some suggesting that **M**_22_(*π*) was primarily an indicator of particle composition (Collister et al. 2018; Dionisi et al. 2020) and others suggesting that it was more sensitive to particle shape and size (Schulien et al. 2020). Particles that contribute to light scattering in natural waters are composed of a diversity of organic and inorganic matrices with a large degree of structural and morphological complexity, making it historically difficult to explore the response of **M**_22_(*π*) to particle shape, size, and composition in silico using models of polarized light scattering. Calculations that resolve this complexity have only recently been developed for a select few marine particles (e.g., coccoliths [Zhai et al. 2013; Bi and Yang 2015], colony‐forming *Microcystis* sp. [Zhai et al. 2020], and chain forming diatoms [Sun et al. 2016]), but the simplifying assumptions required to make them tractable have yet to be validated against light‐scattering measurements at angles relevant to the lidar sampling geometry.

Additionally, oceanographic lidar studies have struggled to account for the influence of multiple scattering on profiles of *δ* (Collister et al. 2018; Schulien et al. 2020). If left unaccounted for, the concentration dependence imparted on *δ* by multiple scattering can result in inconsistent relationships between *δ* and the bulk properties of the particle assemblage, especially in regions of the ocean where particle concentration and bulk characteristics covary. Monte Carlo radiative transfer models have been developed for the purpose of exploring this effect on lidar measurements (Liu et al. 2019), but optical closure studies required to investigate the influence of water column optical properties and system geometry on profiles of *δ* have been difficult to perform given challenges associated with measuring profiles of *δ* from airborne lidar systems and in‐water optical properties at similar time and space scales. Shipboard lidar systems have recently permitted some of the first studies of this kind (Liu et al. 2019), but the Monte Carlo technique used for this purpose is computationally expensive and is of limited utility for exploring single and multiple scattering effects on *δ* across large parameter spaces.

This study combined laboratory, field, and modeling experiments to explore the contribution of multiple scattering and changes in bulk particle properties to measurements of *δ*. Linear depolarization measurements performed in the laboratory for several distinct particle assemblages were used to explore the influence of shape, size, and composition on values of **M**_22_(*π*). A simple bio‐optical model based on the small‐angle solution to the vector lidar radiative transfer equation (Zege and Chaikovskaya 1999; Vasilkov et al. 2001) was parameterized with in situ measurements of water column inherent optical properties, and used to explore the influence of particle concentration and composition on measurements of *δ* using a model sensitivity experiment.

## Scattering measurements

### Particle suspensions

The particulate linear depolarization ratio, *δ*_p_, was measured here for several morphologically and compositionally distinct marine particle assemblages. Three phytoplankton cultures were grown for this purpose: a marine cyanobacterium *Synechococcus* sp. (CCFWC 502; Florida Wildlife Research Institute), a marine centric diatom *Thalassiosira weissflogii* (unknown clone number; National Center for Marine Algae and Microbiota), and a calcifying strain of the coccolithophore *Emiliania huxleyi* (CCMP371; National Center for Marine Algae and Microbiota). All cultures were incubated at 22°C with a 13:11 h light:dark cycle and 60 *μ*mol photons m^−2^ s^−1^ incident irradiance provided by two 40 W fluorescent lamps. *Synechococcus* sp. and *T. weissflogii* were grown in L1 medium, and *E. huxleyi* was grown in L1‐Si/25 medium to promote coccolith production (Guillard and Hargraves 1993). Cells were grown in batch‐cultures and were harvested for measurement toward the end of the exponential phase.

An analog for diatom frustules was prepared using food‐grade diatomaceous earth that consisted of intact diatom frustules and fragmented diatom debris (P.F. Harris Mfg.; SKU: DE‐FG8). A coccolith analog was prepared from reagent‐grade powdered calcite (J.T. Baker). The calcite powder was ground and sifted through a 30 *μ*m sieve prior to being suspended in calcium‐saturated ultrapure water (Barnstead Nanopure®; 18 MΩ). The particle size distribution of the stock calcite suspension was reduced to a median particle diameter of 1.9 *μ*m by allowing the suspension to settle for ~ 15 min in a 500‐mL graduated cylinder and retrieving the upper 400 mL of the suspension.

Particle concentration was determined for each stock suspension using a Neubauer counting chamber; calcified cells and detached coccoliths in the *E. huxleyi* culture were identified using cross‐polarized light microscopy (Olympus BH2 microscope; linear polarizers installed after the illuminator and objective). Particle concentrations for each standard addition were determined using the dilution factor for each addition. Particle sizes were determined from microscope images of each suspension by measuring along the major and minor axes of an aliquot of particles. Note that microscopy could not be used effectively to characterize submicron particles that were likely a component of each of the suspensions. An equivalent spherical diameter corresponding to the average projected area of each particle was determined by applying a particle shape model and using Cauchy's theorem that relates the surface area of a three‐dimensional convex shape to its average projected area in two dimensions. A cylindrical particle model was assumed for *Synechococcus* sp., *T. weissflogii*, and detached coccoliths, and a spherical model was assumed for whole *E. huxleyi* cells and laboratory calcite. For the *E. huxleyi* coccoliths, 0.07 *μ*m was used as the height dimension of the cylindrical model as this dimension was too small to measure for coccoliths using visible light microscopy (Linge Johnsen et al. 2019).

The nonwater beam attenuation coefficient, *c*_spec_, was measured at 532 nm for each standard addition of laboratory calcite using a Shimadzu 2700i spectrophotometer and a 10 cm cuvette. For every other particle suspension, *c*_spec_ was measured for the stock solutions using a 1 cm cuvette, and dilution factors were used to calculate *c*_spec_ for each standard addition to the measurement tank. Measurements of *c*_spec_ from the stock suspensions are potentially biased by multiple scattering due to their high optical densities, but *c*_spec_ served only as scaling factors for plotting the response of *δ*_p_ to changes in particle concentration on a single scale, and it was not used for any other calculations.

### Scattering measurement procedures

**Fig. 1:**
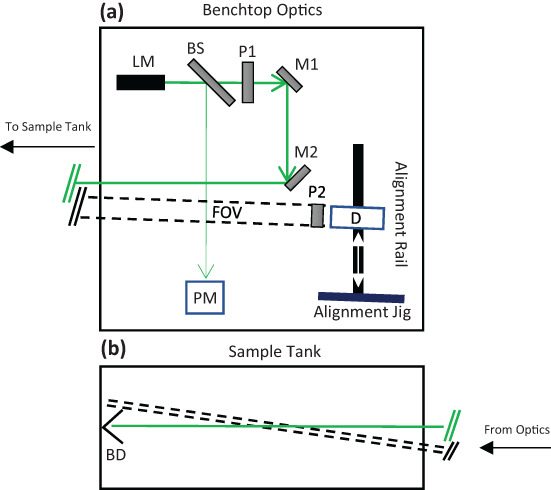
Plan view schematic showing the optical configuration for the depolarization measurement. (**a**) Source optics consisted of a 532 nm laser module (LM), beamsplitter (BS), linear polarizer (P1), and two beam‐steering mirrors (M1 and M2). The detector module (D) consisted of a collecting lens, 0.5 mm aperture, and a photomultiplier tube mounted to an optical rail, allowing it to be translated (white arrows) between an alignment jig (blue bar) and the measurement position. A second linear polarizer (P2) was positioned in front of the detector module to serve as a polarization analyzer. A reference detector (PM) sampled the split beam to measure temporal variations in beam energy. The beam path is shown in green and the field of view is shown by dashed black lines. (**b**) The beam and field of view overlapped in the center of the sample tank, which was positioned downrange from the optical bench. The beam was terminated by a beam dump (BD) positioned at the rear of the tank to prevent specular reflection off the back wall of the aquarium. Drawings are not to scale; angles are exaggerated for illustration purposes.

Linear depolarization was measured at a scattering angle of 178.5° using a modular benchtop laboratory optical assembly (Fig. 1). The light source consisted of a 532 nm collimated solid state laser module (LM; Thorlabs CPS532; 4.5 mW; 3.55 mm diameter; < 0.5 mrad divergence) aligned such that the major polarization axis was parallel to the benchtop reference plane. A fraction of the beam was diverted by a beam sampler, positioned directly after the laser, to a power meter (Thorlabs S130C) that served as a reference detector. A linear polarizer (measured extinction ratio > 250 : 1) positioned after the beam sampler was used to polarize the laser source, and a pair of beam steering mirrors oriented the beam to be orthogonal to the face of a glass aquarium (76 cm × 30 cm × 30 cm) positioned 1 m from the detection optics. A beam dump was positioned within the aquarium, just before the far glass wall, to eliminate specular reflection of the beam.

The receiver assembly consisted of a collecting lens (Thorlabs LA1608; *f* = 75.0 mm), a 0.5 mm aperture at the focal point of the lens, a 532 nm bandpass filter (Semrock LL01‐532‐12.5) to reject ambient light, and a photomultiplier tube (Hamamatsu H10721‐20). The full‐angle receiver field of view was constrained by the collection optics to be 7 mrad. A linear polarizer (measured extinction ratio > 250:1) fixed to an indexed rotation mount at the front of the detector assembly served as a polarization analyzer. A power supply (Keithley 2231A‐30‐3) provided 5 V to the photomultiplier tube module and 0.9 V to the photomultiplier gain control. The photomultiplier output signal was recorded and averaged over 200 ms using an oscilloscope (Tektronix TDS2024C).

Vertical alignment of the detector to the scattering volume was achieved by temporarily placing a diffuse white target in the beam path at the center of the tank and adjusting the height of the detector assembly to maximize the signal. The detector assembly was then set to view at an in‐air angle of 178° (178.5° in‐water) from the source beam using an alignment jig, and was aligned horizontally by translating the detector assembly along a rail mounted behind the mirror assembly until the signal was maximized. Correct alignment was confirmed by viewing the image of the alignment spot projected by the collection lens onto the receiver aperture. The scattering volume sampled by the detector assembly was approximately 7 mL, and occupied the entire 70 cm distance between the front glass and the beam dump.

Depolarization measurements were made on serial additions of scattering material to the aquarium filled with 23 liters of filtered water. For the laboratory calcite measurements, the background consisted of ultrapure water (Barnstead Nanopure®; 18 MΩ) amended with calcium chloride (10 mM) and sodium bicarbonate (2 mM), and buffered with sodium hydroxide to a pH of 8.2 to prevent calcite dissolution. For all measurements involving live phytoplankton and the diatomaceous earth‐mixing experiment, artificial seawater (Instant Ocean®; salinity = 32) filtered through a 0.2‐*μ*m cartridge filter (Pall AcroPak 500) was used in place of pure water to prevent osmotic cell lysis. Filtered water was degassed in the measurement chamber for a 24‐h period prior to each measurement. During this period, the water was recirculated through a 0.2‐*μ*m cartridge filter, and the tank was covered to prevent the accumulation of airborne particles. During each approximately 1‐h measurement period, the recirculation pump was run without the filter to homogenize the sample volume and randomize particle orientation.

The co‐ and cross‐polarized signal returns were measured for the background water, *S*_||_^blank^ and *S*_⊥_^blank^ respectively, and each sample addition, *S*_||_^sample^ and *S*_⊥_^sample^, by rotating the linear polarizer placed in front of the detector between the co‐ and cross‐polarized orientations. Dark counts were measured by obscuring the detector and were subtracted from each measurement. The particulate depolarization ratio, *δ*_p_, was then calculated as:

(3) $$\delta_{p}=\frac{S_{⊥}^{\text{sample}}-S_{⊥}^{\text{blank}}}{S_{∥}^{\text{sample}}-S_{∥}^{\text{blank}}}$$

*δ*_p_ was measured at a series of increasing particle concentrations to confirm that our measurements were uninfluenced by multiple scattering, which would have resulted in a positive relationship between *δ*_p_ and particle concentration. *δ*_p_ was averaged over each serial addition and the value of **M**_22_(178.5°) for each particle assemblage, $M_{22}^{p}$(*π*), was estimated from *δ*_p_ using Eq. 2. Since measurements were not made in the exact backscattering direction, estimates of $M_{22}^{p}\left(178.5{}^{\circ}\right)$ from *δ*_p_ assume that the off‐diagonal Mueller matrix elements had a negligible influence on *δ*_p_ at scattering angles very close to 180° (Voss and Fry 1984; Miffre et al. 2019). For comparison with lidar measurements of *δ* and light‐scattering calculations of $M_{22}^{p}$(*π*), we also assumed that there were no strong variations in $M_{22}^{p}$(*θ*) at angles very close to 180°, such that our measurements at 178.5° closely approximate values in the exact backscattering direction (Miffre et al. 2019). For simplicity, we will refer to our measurements of $M_{22}^{p}$(*θ*) at 178.5° as $M_{22}^{p}$(*π*) throughout the remainder of this work.

For the *E. huxleyi* culture, $M_{22}^{p}$(*π*) was partitioned into an acid‐labile component consisting of attached and detached coccoliths, $M_{22}^{\prime }$(*π*), and an acid‐stable component consisting of unplated cells, $M_{22}^{\text{acid}}$(*π*). This was accomplished at the end of the serial addition by acidifying the water in the aquarium to pH 5.5 using glacial acetic acid to dissolve the calcite, and measuring $M_{22}^{\text{acid}}$(*π*) and the scattering return signal from the acid‐stable particle population, $S_{\text{acid}}$ (Balch et al. 2001). $M_{22}^{\prime }$(*π*) was then calculated by assuming a linear contribution of $M_{22}^{\prime }$(*π*) and $M_{22}^{\text{acid}}$(*π*) to $M_{22}^{p}$(*π*) that was proportional to the contribution of scattering by acid‐labile particles, S′, and acid‐stable particles, $S_{\text{acid}}$, to the scattering return measured for the bulk culture, *S*:

(4) $$M_{22}^{p}\left(\pi \right)=\frac{M_{22}^{\prime }\left(\pi \right)S\prime +M_{22}^{\text{acid}}\left(\pi \right)S_{\text{acid}}}{S}$$

For these measurements, standard additions were continued beyond the initial acidification while maintaining a pH of 5.5 to confirm that the measurements remained uninfluenced by multiple scattering.

Unexpectedly high values of *δ*_p_ measured for *T. weissflogii* prompted us to conduct particle‐mixing experiments at the conclusion of the *T. weissflogii* and diatomaceous earth measurements. *E. huxleyi* culture was added serially at the end of the *T. weissflogii* experiment, and *T. weissflogii* culture was added serially at the end of the diatomaceous earth experiment. A least‐squares linear mixing model was used to estimate $M_{22}^{p}$(*π*) for the added particle suspension from the change in bulk $M_{22}^{p}$(*π*) with each mixing addition. Cultures used for the mixing experiment portions of the *T. weissflogii* and diatomaceous earth measurements were in stationary or senescent phase and left over from the initial light‐scattering experiments. Microscopic examination of the cultures revealed intact cells as well as an accumulation of cellular detritus, so values for $M_{22}^{p}$(*π*) for the mixedin particle suspensions are not necessarily representative of healthy cultures. Nonetheless, the particle‐mixing experiments were useful measurement validation exercises.

## Bio‐optical modeling

### Modeling framework

We constructed a simple bio‐optical model to account for the influence of single and multiple scattering on lidar measurements of *δ* made in the field, and used it to explore the sensitivity of *δ* to changes in particle concentration and bulk particle properties. The model was based on an analytical solution to the lidar radiative transfer equation that uses the small‐angle approximation to solve for the vertical distribution of energy and the polarization characteristics of a backscattered laser pulse (Zege and Chaikovskaya 1999; Vasilkov et al. 2001). For an initially linearly polarized pulse, the depth‐dependent solution for the degree of linear polarization of the return pulse, *g*, takes the form:

(5) $$g\left(z\right)=M_{22}\left(\pi \right)\exp\left(-2\mathrm{\phi bz}\right)$$

where *z* represents distance, **M**_22_(*π*) represents the 2,2‐element of the reduced scattering matrix for whole seawater, *b* is the total scattering coefficient, and *ϕ* is a depolarization factor that controls the exponential decay of *g* with optical depth (*bz*) due to multiple forward scattering. Here, *g* refers to the fraction of the lidar return that retained the linear polarization state of the emitted pulse (Vasilkov et al. 2001). The parameter *ϕ* depends on the shape of **M**_22_(*θ*) in the near‐forward direction and the sampling geometry of the lidar system (Vasilkov et al. 2001). In practice, *ϕ* is treated as a fitting parameter due to challenges associated with measuring **M**_22_(*θ*) in the near‐forward direction and sensitivities of *ϕ* to lidar source and detector geometries that are difficult to characterize (Vasilkov et al. 2001; Chaikovskaya 2006). **M**_22_(*π*) was deconstructed into contributions from *m* scattering components as:

(6) $$M_{22}\left(\pi \right)=\sum_{n=1}^{m}M_{22}^{n}\left(\pi \right)\frac{\beta_{n}\left(\pi \right)}{\beta \left(\pi \right)}\;$$

where $M_{22}^{n}$(*π*) is the 2,2‐element of the normalized scattering matrix element for component *n*, *β*_*n*_(*π*) is the volume scattering by component *n* at *π*, and *β*(*π*) is the volume scattering of the bulk medium at *π*. The forward scattering depolarization parameter was deconstructed in a similar manner as:

(7) $$\phi =\sum_{n=1}^{m}\phi_{n}\frac{b_{n}}{b}$$

where *ϕ*_*n*_ and *b*_*n*_ are the depolarization factor and scattering coefficient for component *n*.

Two model sensitivity experiments were conducted to explore the role of particle type and multiple scattering in measurements of *δ*. For the first experiment, we tested whether patterns in *δ* could be explained by assuming a single particle type. **M**_22_(*π*) was parameterized as

(8) $$M_{22}\left(\pi \right)=\frac{1}{2\pi \beta \left(\pi \right)}\left[M_{22}^{p}\left(\pi \right)\frac{b_{\mathrm{bp}}}{\chi_{p}\left(\pi \right)}+M_{22}^{w}\left(\pi \right)\frac{b_{\mathrm{bsw}}}{\chi_{\mathrm{sw}}\left(\pi \right)}\right]$$

where the *χ*(*π*) factors convert between total hemispherical backscatter and backscatter at *π* for the particulate and pure seawater components respectively, *b*_bp_ is the particulate backscattering coefficient, *b*_bsw_ is the backscattering coefficient for pure seawater, and *β*(*π*) is the total volume scattering coefficient at *π* (derived from the sum of *β*(*π*) for each individual component). *ϕ* was parameterized for these components using Eq. 7:

(9) $$\phi =\phi_{p}\frac{b_{b}}{b}+\phi_{\mathrm{sw}}\frac{b_{\mathrm{bsw}}}{b}$$

where *b*_p_ and *b*_sw_ are the scattering coefficients for particles and pure seawater respectively. For the second experiment, we explored the influence of scattering by coccoliths on **M**_22_(*π*) by assuming three distinct scattering populations: (1) acid‐labile particles [$M_{22}^{\prime }$(*π*)], (2) acid‐stable particles [$M_{22}^{\text{acid}}$(*π*)], and (3) pure seawater [$M_{22}^{\mathrm{sw}}$(*π*)]. Substituting these into Eq. 6 gives:

(10) $$M_{22}\left(\pi \right)=\frac{1}{2\pi \beta \left(\pi \right)}\left[M_{22}^{\prime }\left(\pi \right)\frac{b_{b}\prime }{\chi \prime \left(\pi \right)}+M_{22}^{\text{acid}}\left(\pi \right)\frac{b_{\mathrm{bp}}-b_{b}\prime }{\chi_{\text{acid}}\left(\pi \right)}+M_{22}^{\mathrm{sw}}\left(\pi \right)\frac{b_{\mathrm{bsw}}}{\chi_{\mathrm{sw}}\left(\pi \right)}\right]$$

where the *χ*(*π*) factors convert between total hemispherical backscatter and *π* backscatter for the acid‐labile, acid‐stable, and pure seawater components respectively. *ϕ* was parameterized for these three components using Eq. 7:

(11) $$\phi =\phi^{\prime }\frac{b\prime }{b}+\phi_{\text{acid}}\frac{b_{\text{acid}}}{b}+\phi_{\mathrm{sw}}\frac{b_{\mathrm{sw}}}{b}$$

where *b*′ is the scattering coefficient for acid‐labile particles, *b*_acid_ is the scattering coefficient for acid‐stable particles, *b*_acid_ = *b*_p_ – *b′*, and *b*_sw_ is the scattering coefficient for seawater.

### Model parameterization

The model was parameterized using a dataset of in situ inherent optical properties that were collected concurrently with oceanographic lidar measurements of *δ* made during the CoccoMix research expedition in the North Atlantic (*see* Collister et al. 2020 for more details of the cruise). For the duration of the expedition, *δ* was measured at a distance along the beam of 6.5 m from the sea surface using a shipboard lidar system mounted at the bow of the ship and positioned at an angle of 35° from nadir. An underway flow‐through system was used to sample water continuously from the ship's seawater intake at 5 m depth, and a WetLABS *ac*‐9 spectrophotometer was plumbed into the system to measure the nonwater absorption and attenuation coefficients, *a*_pg_ and *c*_pg_ respectively, throughout the expedition. The particulate scattering coefficient, *b*_p_, was calculated as *b*_p_ = *c*_pg_ – *a*_pg_ by assuming that the scattering coefficient for the dissolved fraction (*b*_g_) was zero, and *b* was calculated as *b* = *b*_p_ + *b*_sw_, where *b*_sw_ is the scattering coefficient for pure seawater calculated from surface measurements of temperature and salinity (Zhang et al. 2009). The particulate backscattering coefficient, *b*_bp_, was measured using a Wyatt EOS light‐scattering detector, and the acid‐labile backscattering coefficient, *b*_b_′, was measured as the difference between total *b*_bp_ and measurements of backscattering from a sample that was acidified to dissolve all particulate calcite. The total backscattering coefficient was calculated as *b*_b_ = *b*_bp_ + *b*_bsw_, where *b*_bsw_ was also calculated from surface measurements of temperature and salinity (Zhang et al. 2009).

Values used to parameterize the lidar depolarization model are summarized in Table 1. Backscattering coefficients in Eqs. 8 and 10, and scattering coefficients in Eqs. 9 and 11, were parameterized for each component using the in situ measurements described above. *b*′ was parameterized from measurements of *b*_b_′ by assuming a constant backscattering ratio of 0.025 for coccoliths (Voss et al. 1998). We assumed a value of 0.5 for *χ*_p_(*π*), *χ′(π*), and *χ*_acid_(*π*), and a value of 0.68 for *χ*_sw_(*π*) (Zhang et al. 2009; Schulien et al. 2017). $M_{22}^{w}$(*π*) and *ϕ*_sw_ were set to 1 and 0 respectively since molecular scattering by water is weakly depolarizing (Zhang et al. 2019). In the first experiment, two free‐parameters remained, $M_{22}^{p}$(*π*) and *ϕ*_p_. The model was solved for values of $M_{22}^{p}$(*π*) that ranged from 0.5 to 1 and values of *ϕ*_p_ that ranged from 0 to 0.4. For the second experiment, $M_{22}^{\prime }$(*π*) was parameterized from laboratory measurements of depolarization by the acid labile fraction of the *E. huxleyi* culture (0.78), leaving three free parameters in the model: $M_{22}^{\text{acid}}$(*π*), *ϕ*′, and *ϕ*_acid_. A model sensitivity analysis was performed by solving for *g* using values of $M_{22}^{\text{acid}}$(*π*) ranging from 0.5 to 1, and values of *ϕ*_acid_ and *ϕ*′ ranging from 0 to 0.4. Model predictions of *δ* were computed from *g* as:

(12) $$\delta =\frac{1-g}{1+g}$$

and compared with field measurements of *δ* using root‐mean‐square error (RMSE):

(13) $$\text{RMSE}=\sqrt{\frac{\sum_{i=1}^{N}{\left(\delta_{i}-{\hat{\delta }}_{i}\right)}^{2}}{N-k}}$$

where $\delta_{i}$ and ${\hat{\delta }}_{i}$ are the modeled and measured values of the linear depolarization ratio for the *i*th observation, *N* is the total number of observations, and *k* is the number of explanatory coefficients included in the model.

**Table 1:** Model parameterizations.

Parameter	Values	Units	Source
Single‐particle experiment			
$M_{22}^{\mathrm{sw}}$(*π*)	1	Dimensionless	Zhang et al. (2009)
$M_{22}^{p}$(*π*)	0.5–1	Dimensionless	Free parameter
*ϕ*_sw_	0	Dimensionless	Zhang et al. (2009)
*ϕ*_p_	0–0.4	Dimensionless	Free parameter
*χ*_sw_	0.68	sr	Zhang et al. (2009)
*χ*_p_	0.5	sr	Schulien et al. (2017)
Two‐particle experiment			
$M_{22}^{\mathrm{sw}}$(*π*)	1	Dimensionless	Zhang et al. (2009)
$M_{22}^{\prime }$(*π*)	0.78	Dimensionless	Table 2
$M_{22}^{\text{acid}}$(*π*)	0.5–1	Dimensionless	Free parameter
*ϕ*_sw_	0	Dimensionless	Zhang et al. (2009)
*ϕ*′	0–0.4	Dimensionless	Free parameter
*ϕ*_acid_	0–0.4	Dimensionless	Free parameter
*χ*_sw_	0.68	sr	Zhang et al. (2009)
*χ*_p_ = *χ*′ = *χ*_acid_	0.5	sr	Schulien et al. (2017)
*b*_b_′/*b*′	0.025	Dimensionless	Voss et al. (1998)

## Results

### Particle characteristics

**Fig. 2:**
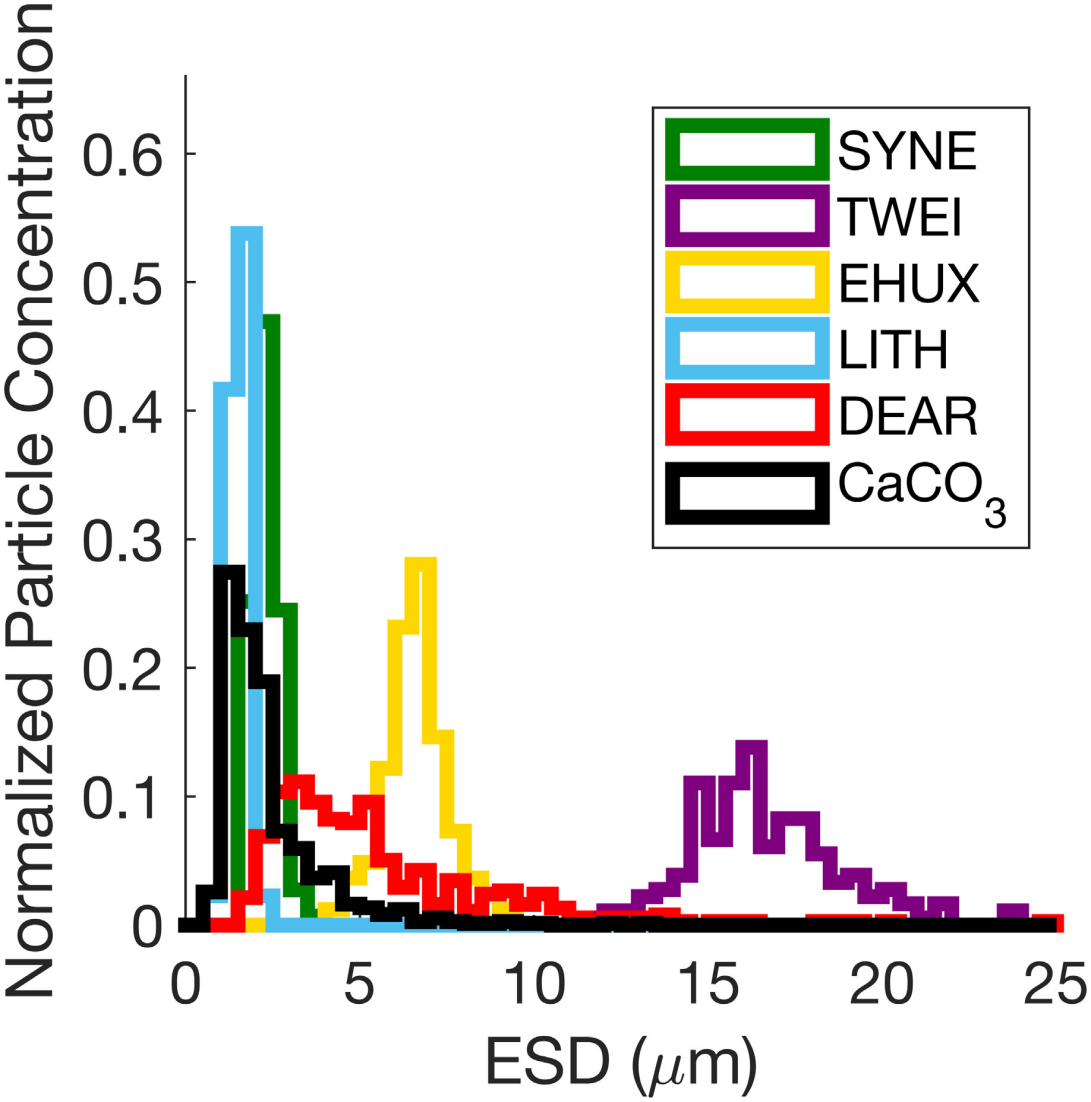
Histograms showing particle size distributions, expressed as equivalent spherical diameter (ESD), for each particle suspension. *Synechococcus* sp. (green; SYNE), *T. weissflogii* (purple; TWEI), *E. huxleyi* (gold; EHUX), *E. huxleyi* coccoliths (cyan; LITH), diatomaceous earth (red; DEAR), and laboratory calcite (black; CaCO_3_). Particle size distributions were normalized such that each histogram sums to one.

**Fig. 3:**
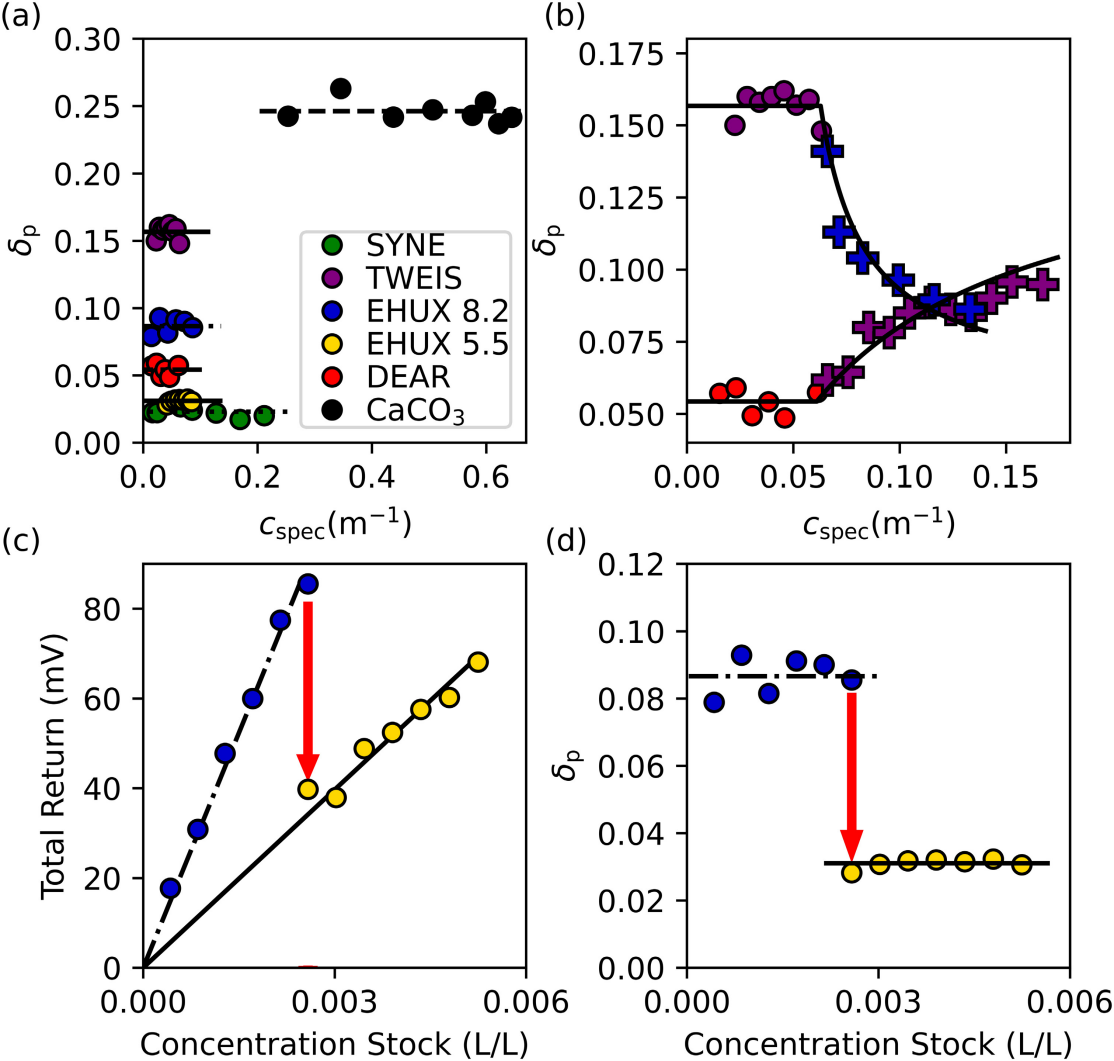
Laboratory depolarization experiment results. (**a**) Plot of the particulate depolarization ratio (*δ*_p_) vs. the beam attenuation coefficient (*c*_spec_) for each experiment (*Synechococcus* sp. [green; SYNE], *T. weissflogii* [purple; TWEI], *E. huxleyi* at pH 8.2 [blue; EHUX 8.2], *E. huxleyi* at pH 5.5 [gold; EHUX 5.5], diatomaceous earth [red; DEAR]), and laboratory calcite (black, CaCO_3_). (**b**) Results from the particle‐mixing experiments. Marker colors are consistent with the legend in (**a**) and mixing additions are plotted as “pluses.” Black lines represent least‐squares fits to a linear mixing model used to estimate the **M**_22_(*π*) for the added particle suspension. (**c**) Total return signal and (**d**) *δ*_p_ from the *E. huxleyi* acidification experiment plotted against the concentration of stock algal culture. Red arrows highlight the change in the total return signal and *δ*_p_ after acidification.

*Synechococcus* sp. cells were cylindrical with a mean aspect ratio of 3.6, and a mean diameter of 2.3 ± 0.38 *μ*m (Fig. 2; Supporting Information Fig. S1a; Table 2). *T. weissflogii* cells were cylindrical with a mean aspect ratio of 2.1, and were the largest particles measured here with a mean diameter of 16.7 ± 2.2 *μ*m (Fig. 2; Supporting Information Fig. S1b; Table 2). The diatomaceous earth suspension consisted of intact diatom frustules that were similar in shape to the live *T. weissflogii* cells, as well as small silica debris (Supporting Information Fig. S1c). The size distribution of the diatomaceous earth suspension was right skewed with a median diameter of 4.6 *μ*m and a measured range between 2 and 25 *μ*m (Fig. 2, Table 2). The *E. huxleyi* culture was composed of detached coccoliths and calcified spherical cells with a free‐coccolith to calcified cell ratio of 13:1 (Supporting Information Fig. S1d). All cells had intact coccospheres at pH 8.2, and acidification of the culture to pH 5.5 resulted in complete dissolution of suspended and attached coccoliths that was confirmed by cross‐polarized microscopy. The size distribution of calcified *E. huxleyi* cells was approximately normal with a mean diameter of 6.6 ± 0.88 *μ*m (Fig. 2; Table 2). Detached coccoliths were the smallest particles measured with a mean diameter of 1.5 ± 0.24 *μ*m. The size distribution of the powdered laboratory calcite suspension was right skewed, with a median diameter of 1.9 *μ*m and a measured range between 1 and 13 *μ*m (Fig. 3; Supporting Information Fig. S1e; Table 2).

**Table 2:** Morphological and optical characteristics of particle suspensions used in light‐scattering experiment.

Particle	Shape model	Equivalent spherical diameter (*μ*m)	Average particle aspect ratio	Undiluted stock particle concentration (particles mL^−1^)	Particulate depolarization ratio, *δ*_p_ (± 95% CI)	$M_{22}^{p}$ (± 95% CI)
*Synechococcus* sp.	Cylinder	2.3	3.6	1.7×10^9^	0.023(0.003)	0.96(0.005)
*T. weissflogii*	Cylinder	16.7	2.1	3.2×10^6^	0.16(0.004)	0.73(0.005)
*E. huxleyi*						
Naked cells	Sphere	‐	1	9.7×10^5^	0.031(0.005)	0.94(0.008)
Coccoliths	Cylinder	1.5	‐	1.3×10^7^	0.12(0.001)	0.78(0.002)
Cells + coccospheres	Sphere	6.6	1	9.7×10^5^	0.087(0.001)	0.84(0.002)
Diatomaceous earth	Cylinder	4.6^a^	1.5	7.4×10^7^	0.054(0.004)	0.90(0.006)
Laboratory calcite	Sphere	1.9^a^	1	3.6×10^7^	0.25(0.006)	0.60(0.008)

^a^ Median equivalent spherical diameters are presented for non‐normal particle size distributions. All other equivalent spherical diameters are presented are averages.

### Scattering measurements

Measured values of *δ*_p_ were independent of particle concentration, providing confidence that measurements of *δ*_p_ were not influenced by multiple scattering (Fig. 3a). *δ*_p_ ranged from a minimum of 0.023 for *Synechococcus* sp. to a maximum value of 0.25 for the laboratory calcite suspension (Fig. 3a). Values of *δ*_p_ measured for *T. weissflogii* (*δ*_p_ = 0.16) were unexpectedly high relative to measurements of *δ*_p_ for the diatomaceous earth suspension (*δ*_p_ = 0.054) that contained diatom frustules similar in morphology and composition to those of the living diatom, as well as the coccolithophore culture (*δ*_p_ = 0.087) that contained a large concentration of birefringent and high refractive index calcite coccoliths. Measurements of *δ*_p_ from the particle‐mixing experiments confirmed the elevated depolarization by *T. weissflogii* relative to *E. huxleyi* and diatomaceous earth because *δ*_p_ decreased asymptotically with the addition of *E. huxleyi* culture to the *T. weissflogii* experiment and increased asymptotically with the addition of *T. weissflogii* culture to the diatomaceous earth experiment (Fig. 3b). A least‐squares linear mixing model predicted that the *E. huxleyi* and *T. weissflogii* cultures used in the mixing experiment were somewhat less depolarizing than the corresponding healthy cultures, with a value of *δ*_p_ = 0.15 for *T. weissflogii* (compared to *δ*_p_ = 0.16 for the healthy culture) and *δ*_p_ = 0.058 for *E. huxleyi* (compared to *δ*_p_ = 0.087 for the healthy culture). For the *E. huxleyi* acidification experiment, the slope of *S* vs. the concentration of stock particle solution decreased from 3.50 × 10^4^ to 1.32 × 10^4^ mV L‐seawater L‐stock^−1^ when the pH was lowered to 5.5, suggesting that calcite contributed to 62% of the scattered flux at *π* for the coccolithophore culture (Fig. 3c). After the sample chamber was acidified, *δ*_p_ of *E. huxleyi* decreased from 0.087 to 0.031 (Fig. 3d).

Small phytoplankton lacking biomineralized shells were the least depolarizing, with *Synechococcus* sp. having an $M_{22}^{p}$(*π*) value of 0.96 and the acidified *E. huxleyi* culture having an $M_{22}^{p}$(*π*) value of 0.94 (Table 2). $M_{22}^{p}$(*π*) was not a strong predictor of shape for these small, optically soft particles as both particle suspensions were weakly depolarizing despite the strong deviation of *Synechococcus* sp. cell shape from sphericity (Supporting Information Fig. S1; Table 2). $M_{22}^{p}$(*π*) for the acid‐labile fraction of *E. huxleyi* was 0.78, with the presence of coccoliths decreasing the value of $M_{22}^{p}$(*π*) for the bulk culture from 0.94 for decalcified cells to 0.84 for a mixture of free coccoliths and cells with intact coccospheres (Table 2). The laboratory calcite suspension was a stronger depolarizer than coccolith calcite, with an $M_{22}^{p}$(*π*) value of 0.60 that was substantially lower than any of the particles measured here. *T. weissflogii* was the most depolarizing of the phytoplankton measured, with an $M_{22}^{p}$(*π*) value of 0.73. The suspension of diatomaceous earth was less depolarizing than the *T. weissflogii* culture, with an $M_{22}^{p}$(*π*) value of 0.90.

### Model sensitivity analysis

**Fig. 4:**
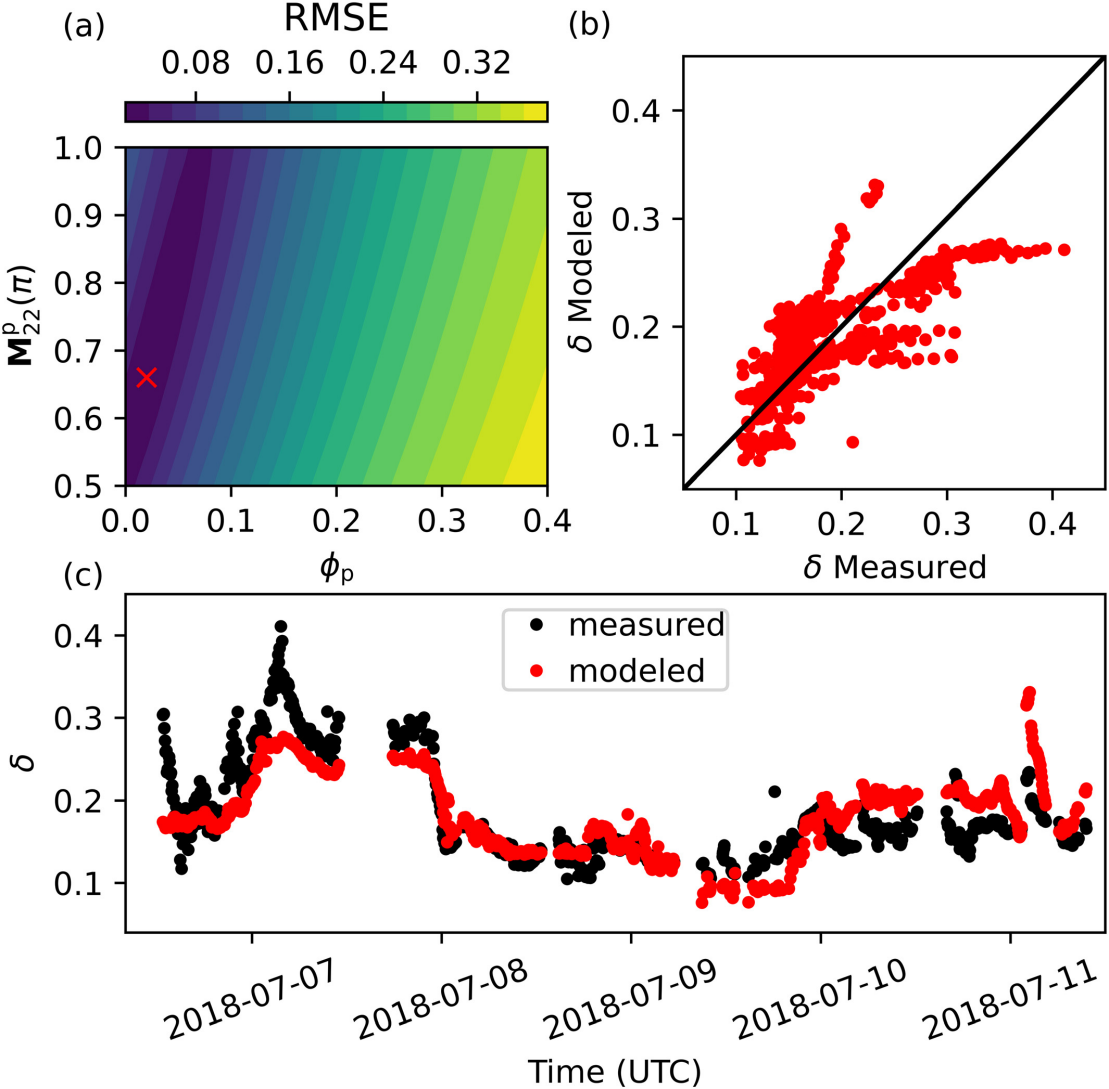
Plots showing results from the single‐particle sensitivity experiment. (**a**) Contours of RMSE comparing modeled and measured values of *δ* are plotted as a function of model inputs $M_{22}^{p}$(*π*) and *ϕ*_p_. The red “x” highlights the model input parameters that minimize RMSE. (**b**) Values of *δ* from the RMSE optimized solution plotted against measured values of *δ* (RMSE = 0.023, $M_{22}^{p}$ = 0.66, *ϕ*_p_ = 0.02). (**c**) Model estimates of *δ* (red) from the RMSE optimized model solution plotted as a time series alongside lidar measurements of *δ* from Collister et al. (2020).

**Fig. 5:**
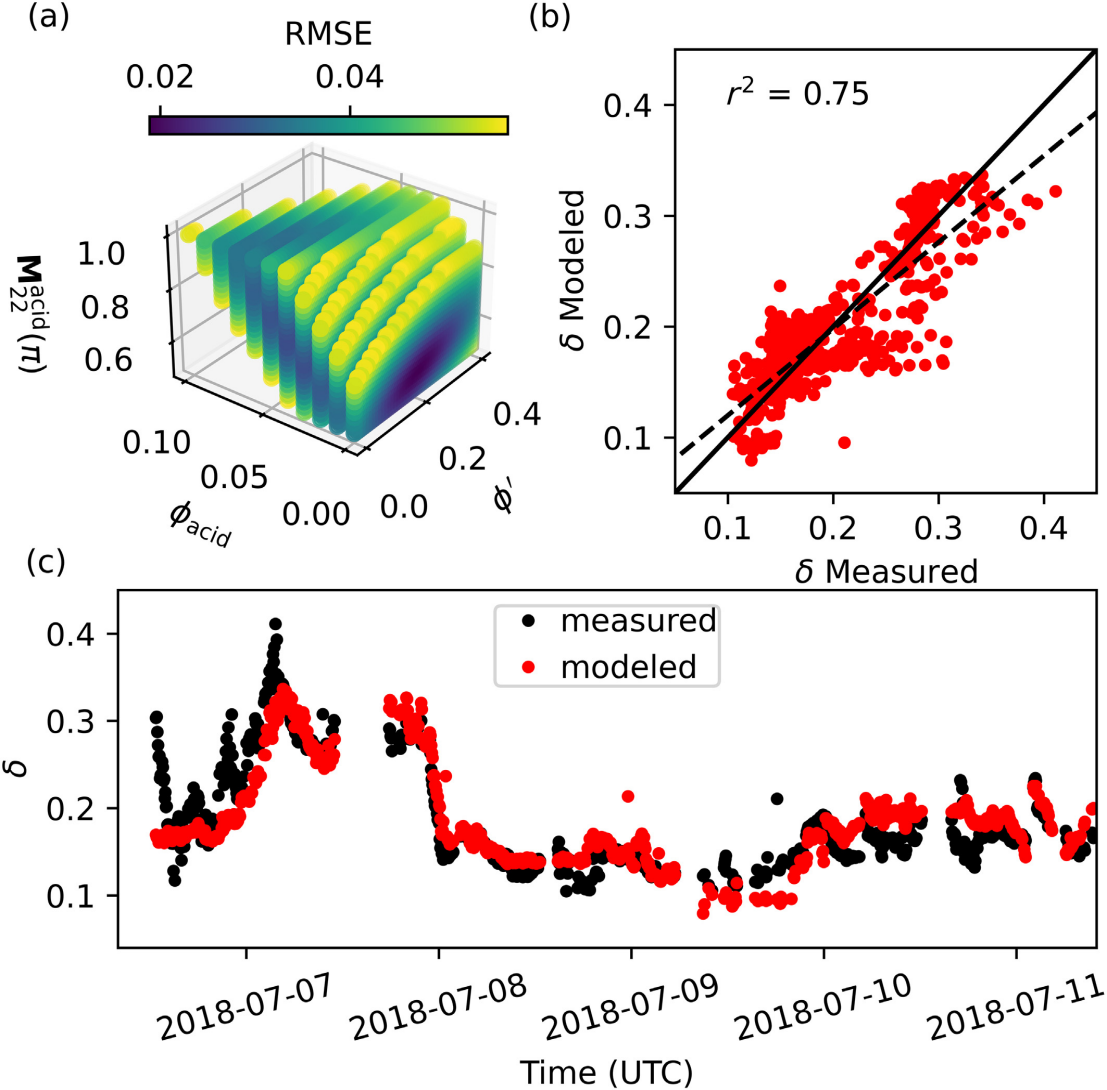
Plots showing results from the two‐particle model sensitivity experiment. (**a**) The location of each point represents the value of model inputs *ϕ*′, *ϕ*_acid_, and $M_{22}^{\mathrm{POC}}$(*π*). The color map shows the corresponding value of RMSE comparing modeled and measured values of *δ*. (**b**) Modeled values of *δ* from the RMSE optimized model solution (RMSE = 0.019, $M_{22}^{\mathrm{POC}}$ = 0.58, *ϕ*_acid_ = 0.0, *ϕ*′ = 0.19) plotted against measured values of *δ* from Collister et al. (2020). The solid line shows the 1:1 relationship, and the dashed line shows the results of a least‐squares linear regression (slope = 0.78 ± 0.031 [95% confidence interval]; intercept = 0.042 ± 0.006 [95% confidence interval]; *r* ^2^ = 0.75, df = 783; SSE = 0.58). (**c**) Model estimates of *δ* (red) from the RMSE optimized model solution plotted as a time series alongside lidar measurements of *δ* from Collister et al. (2020).

Increases in single scattering depolarization (i.e., decreases in $M_{22}^{p}$(*π*)) were compensated for by decreases in depolarization due to multiple scattering (i.e., a decrease in *ϕ*_p_), resulting in contours of RMSE that were elongated in the positive $M_{22}^{p}$(*π*) vs. *ϕ*_p_ direction (Fig. 4a). The optimum model solution with respect to RMSE reproduced many of the broad‐scale patterns found in measurements of *δ*, with values generally tracking patterns in *b* across the entire time series (cf. Fig. 4b,c with fig. 2 in Collister et al. 2020). However, the single‐particle model underestimated *δ* in the region where scattering was strongly coupled to calcite concentration, and overestimated *δ* in the region of strong scattering near the coast where *b*_bp_ and *b*_b_′ became decoupled (Fig. 5b,c).

The optimum solution to the two‐particle model resulted in a minor improvement in RMSE over that of the optimum single‐particle model solution (0.019 vs. 0.023). However, the two‐particle model was better able to capture the magnitude of *δ* across the entire cruise track, characterized by a coccolithophore bloom on Georges Bank to a mix of coccolithophore bloom and coastally influenced waters in the New York Bight (Figs. 4b,c, 5b,c; Collister et al. 2020). For the two‐particle model, the RMSE for all simulations with a value of *ϕ*_acid_ > 0.1 was > 0.057 (i.e., three times the RMSE for the optimum solution; Fig. 5a). The two‐particle model was less sensitive to the parameterization of *ϕ*′, and solutions with RMSE less than 0.057 existed over the entire range of *ϕ*′ and $M_{22}^{\text{acid}}$(*π*) values considered here (0–0.4). Increasing values of *ϕ*_acid_ resulted in optimum values of *ϕ*′ and $M_{22}^{\text{acid}}$(*π*) that were inversely related, where a decrease in $M_{22}^{\text{acid}}$(*π*) (i.e., an increase in the backscattering depolarization by the acid‐stable particle population) was compensated for by an increase in *ϕ*′ (i.e., increase in the forward scattering depolarization by coccolith calcite, Fig. 5a). For values of *ϕ*_acid_ greater than 0.1, optimum values of $M_{22}^{\text{acid}}$(*π*) and *ϕ*′ were constrained to their maximum and minimum values respectively, resulting in a rapid decrease in model fitness with increasing *ϕ*_acid_ (Fig. 5a).

**Fig. 6:**
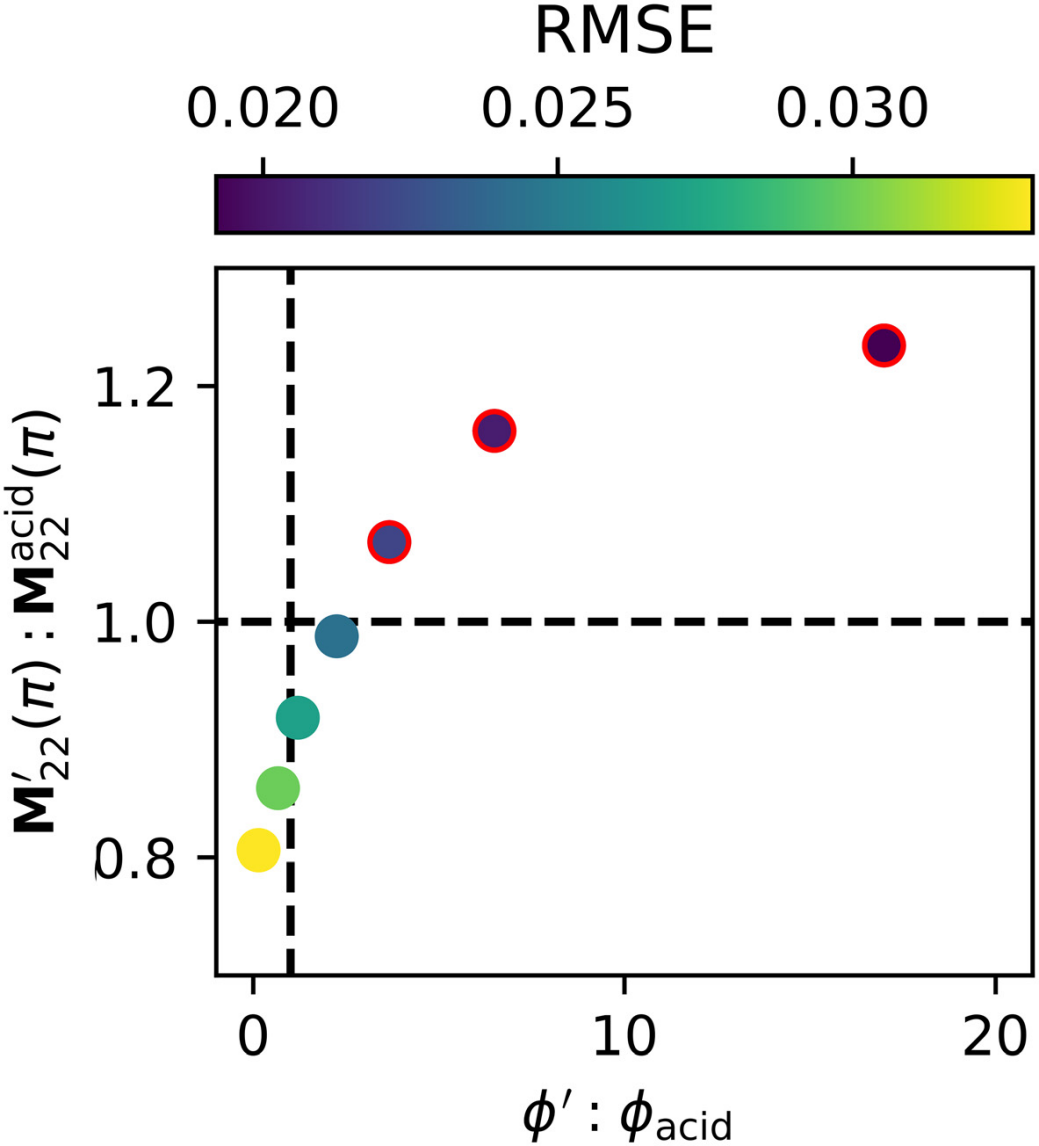
Plot of $M_{22}^{\prime }\left(\pi \right):M_{22}^{\text{acid}}\left(\pi \right)$ vs. *ϕ*′ : *ϕ*_acid_ for nonzero values of *ϕ*_acid_ that resulted in a non‐zero optimum value of *ϕ*′. The color map shows the value of RMSE for each model solution, and symbols outlined in red denote solutions that offer improvements in RMSE relative to the single‐particle model.

For the two‐particle model, RMSE increased with decreasing values of $M_{22}^{\prime }\left(\pi \right):M_{22}^{\text{acid}}\left(\pi \right)$ and *ϕ*′:*ϕ*_acid_ (Fig. 6). Decreases in the forward scattering depolarization by coccolith calcite relative to the acid‐stable particle population were compensated for by an increase in the single scattering depolarization of calcite relative to the background (Fig. 6). The two‐particle model offered improvements in RMSE over the single particle model only within the parameter space where calcite was more depolarizing in the forward direction than the background, acid‐stable particle population (i.e., *ϕ*′:*ϕ*_acid_ > 1). All optimum model solutions where *ϕ*′:*ϕ*_acid_ < 1 required that coccolithophore calcite was more depolarizing in the backward direction (i.e., $M_{22}^{\prime }\left(\pi \right):M_{22}^{\text{acid}}\left(\pi \right)$ < 1) than the acid‐stable particle population. Importantly, there were no optimum model solutions where the acid‐stable particle population was a stronger depolarizer than the acid‐labile particle population in both the forward and backward directions.

## Discussion

The results presented here show that laser backscatter measurements of *δ* exhibit complex and sometimes counterintuitive dependencies on particle size, shape, composition, and concentration that will complicate efforts to use polarized oceanographic lidar as a tool for characterizing particles in the ocean. Laboratory measurements of $M_{22}^{p}\left(\pi \right)$ for several morphologically and compositionally distinct marine particles exhibited a large degree of variability, supporting the idea that spatiotemporal gradients in marine particle characteristics can be detected in lidar measurements of depolarization. However, the behavior of $M_{22}^{p}$(*π*) with respect to particle size, shape, and composition was complex, and $M_{22}^{p}$(*π*) was not a straight‐forward predictor of any single particle intensive property. Modeling results suggest that particle concentration can be a dominant source of variability in lidar measurements of *δ* through the influence of multiple scattering and shifts in the relative contribution of particulate vs. molecular scattering to the lidar return signal. The influence of molecular scattering by seawater on *δ* can be accounted for in a straightforward manner by modeling the contribution of *β*_sw_ to total *β*, but correcting for the influence of multiple scattering on *δ* requires information on the depth distribution of *b* and the shape of **M**_22_(*θ*) in the forward direction that cannot be retrieved independently from the lidar signal.

Although laboratory measurements of $M_{22}^{p}$(*π*) were not driven predominantly by any single particle intensive property, the behavior of $M_{22}^{p}$(*π*) with respect to particle shape, size, and composition agreed qualitatively with theoretical models of polarized light scattering by nonspherical particles. Small, optically soft particles approaching the Rayleigh–Gans limit are expected to be weak depolarizers, with $M_{22}^{p}$(*π*) having a muted sensitivity to particle shape (Mishchenko et al. 2002; Mukherjee et al. 2018). This is consistent with observations of $M_{22}^{p}$(*π*) made here for small, low refractive index phytoplankton cells. *Synechococcus* sp. and decalcified *E. huxleyi* were weak depolarizers, and large deviations of *Synechococcus* sp. from sphericity did not result in a reduced value of $M_{22}^{p}$(*π*) relative to the more spherical *E. huxleyi* cells. As particle size increases into the resonant scattering domain where particle dimensions are comparable to the wavelength of incident light, nonspherical light‐scattering simulations predict an increase in depolarization and an increase in the influence of particle composition on $M_{22}^{p}$(*π*) (Mishchenko et al. 2002; Mukherjee et al. 2018). The influence of particle size on $M_{22}^{p}$(*π*) could account for the large differences in $M_{22}^{p}$(*π*) measured for the laboratory‐grade calcite powder and the coccolith calcite, which had similar compositions but large differences in their particle size distributions.

The *T. weissflogii* culture produced an unexpectedly strong depolarization response, with a value of $M_{22}^{p}$(*π*) that was substantially lower than the compositionally and morphologically similar suspension of diatomaceous earth. One possible explanation for this behavior of $M_{22}^{p}$(*π*) is related to differences in the size distributions of the two diatom‐derived suspensions. Although intact diatomaceous earth frustules were similar in shape and size to the *T. weissflogii* frustules, small silica debris were a large component that reduced the median diameter of the diatomaceous earth suspension (4.6 *μ*m) relative to the live culture (16.7 *μ*m), potentially resulting in elevated values of $M_{22}^{p}$(*π*) for the bulk suspension. The influence of particle size on $M_{22}^{p}$ (*π*) could also explain why *T. weissflogii* was a stronger depolarizer than the birefringent and highly refractive coccoliths, given that the live diatom cells were larger than coccoliths by more than a factor of eight and that coccoliths likely occupied the size sensitive domain of $M_{22}^{p}$(*π*) (Zhai et al. 2013; Bi and Yang 2015). The presence of high refractive index intracellular structures within the live diatom cells but lacking in the diatomaceous earth frustules also could have contributed to enhanced depolarization by *T. weissflogii*. Intracellular structures can play an important role in determining the backscattering efficiency of phytoplankton, but very little is known about their influence on the polarized light‐scattering properties of marine particles (Whitmire et al. 2010; Zhou et al. 2012). Manipulative depolarization experiments that control for particle size or the presence/absence of intracellular structures would be highly informative with respect to these hypotheses.

*E. huxleyi* coccoliths are among the few morphologically complex marine particles for which polarized light‐scattering calculations have been performed. Models predict a wide range of values for $M_{22}^{p}$(*π*) (0.67–0.98) that exhibit sensitivities to particle size and morphology (Zhai et al. 2013; Bi and Yang 2015). The measurement of $M_{22}^{p}$(*π*) for *E. huxleyi* coccoliths was well within the range of values predicted by Zhai et al. (2013), but was more depolarizing than values predicted by Bi and Yang (2015), which returned a minimum value of 0.86. Given that the Bi and Yang (2015) invariant imbedding T‐matrix method validated well against the discrete dipole approximation technique used by Zhai et al. (2013), differences between the two studies likely resulted from the use of morphologically distinct coccolith models. Coccolith morphologies are species‐specific and highly diverse, and the sensitivity of these calculations to subtle differences in the coccolith model geometry likely translates into large interspecies variability in the relationships between $M_{22}^{p}$(*π*), *b*_b_′/*b*_b_, and calcite concentration (Gordon and Du 2001). This sensitivity of $M_{22}^{p}$(*π*) to coccolith geometry may further complicate efforts to develop polarization‐based lidar retrievals of calcite concentration, but it could present an opportunity to use species‐specific relationships between calcite concentration and *δ*_p_ to distinguish monospecific *E. huxleyi* blooms from those with higher coccolithophore diversity.

The interacting effects of particle shape, size, and composition on $M_{22}^{p}$ will complicate efforts to retrieve particle intensive properties from oceanographic lidar measurements of *δ*. For natural particle populations, the sensitivity of $M_{22}^{p}$(*π*) to multiple particle intensive properties is likely to result in regionally specific behaviors of $M_{22}^{p}$(*π*) that depend on local modes of particle variability. In coastal waters where high refractive index minerals and organic detritus are important contributors to backscattering and particle composition is highly variable, $M_{22}^{p}$(*π*) may be driven predominantly by changes in bulk particle refractive index (Twardowski et al. 2001). In the open ocean, where bulk refractive index is often less dynamic, shifts in particle shape and size may be the dominant source of variability in $M_{22}^{p}$(*π*) (Twardowski et al. 2001). Additionally, natural particle assemblages occupy a broader spectrum of sizes, shapes, and compositions than the laboratory generated particle assemblages measured here. The wide range of particle characteristics represented in natural particle assemblages, combined with the interacting effects of particle shape, size, and composition on $M_{22}^{p}$(*π*) can result in an ambiguous response of $M_{22}^{p}$(*π*) to changes in bulk particle characteristics (e.g., particle size distribution slope, average particle aspect ratio, and bulk refractive index). For instance, morphological shifts that occur at opposite ends of the particle size spectrum will have very different effects on $M_{22}^{p}$(*π*), even if they result in identical changes to some bulk particle shape metric. Particle intensive property retrievals that combine polarized oceanographic lidar and passive ocean color or polarimetry could help to constrain some of these ambiguities by providing independent estimates of particle characteristics and light‐scattering information at angles that are inaccessible to the lidar sampling geometry (Ibrahim et al. 2016; El‐Habashi et al. 2021).

The model results presented here suggest that multiple scattering and shifts in the relative contribution of particulate vs. molecular scattering can play a dominant role in controlling patterns in lidar measurements of *δ* from bulk seawater. This was the case for the CoccoMix expedition (Collister et al. 2020), where a single particle model for **M**_22_(*π*) and *ϕ* accounted for as much as 86% of the variance in *δ*. These results suggest that the strong correlation between *δ* and *b*_b_′/*b*_b_ within the coccolithophore bloom was driven predominantly by the covariation between calcite concentration and particulate backscatter, rather than by coccoliths having a substantially lower value of $M_{22}^{p}$(*π*) relative to the acid‐stable particle population. This is consistent with Collister et al. (2020), where a statistical model applied to these data predicted no substantial increase in *δ* with *b*_b_′/*b*_b_ at small optical depths. However, despite resolving much of the variability in *δ* throughout the CoccoMix expedition, a single particle model of depolarization could not reproduce the behavior of *δ* when backscattering became uncoupled from scattering by calcite. The two‐particle model accounted for this bifurcation with several configurations of particle depolarization characteristics, but patterns in RMSE for these solutions point to this behavior resulting from calcite being a stronger depolarizer in the forward direction than particles that composed the acid‐stable fraction of the particle assemblage. This is consistent with observations of strong forward depolarization that can be used to identify birefringent calcite particles using polarized light microscopy, flow‐cytometry, and transmissometry (Balch et al. 1999; Guay and Bishop 2002). Light‐scattering measurements similar to those conducted by Koestner et al. (2020) would be useful for further quantifying the influence of forward scattering by birefringent particles on lidar measurements of *δ* if they were extended to smaller angles (i.e., angles less than ~ 10°).

Previous studies involving polarized oceanographic lidar have struggled to separate the effects of single and multiple scattering on *δ*. Schulien et al. (2020) used the ratio *δ*:*b*_bp_, where *b*_bp_ is estimated from lidar measurements of *β*(*π*), to account for the influence of multiple scattering contained in *δ*. However, this ratio primarily reflects changes in the relative contribution of particulate scattering to the total return signal, and it does not account for the depth dependence of multiple scattering. Changes in this ratio are difficult to interpret, as they can occur by several mechanisms, including changes in $M_{22}^{p}$(*π*), the shape of the scattering phase function, and the depth dependence of multiple scattering. Collister et al. (2020) accounted for the depth dependence of multiple scattering by examining patterns of *δ* as a function of scattering optical depth, but had to resort to an empirical statistical model to separate out contributions of scattering from different components. The model presented here provides a generic framework that can be used to account for multiple scattering and shifts in particle composition in measurements of *δ*, given that *b* can be estimated or measured directly alongside of *δ*. Since *b* cannot be directly retrieved using lidar, routine application of this technique will require either bio‐optical models, in situ measurements, or the development of techniques for retrieving *b* from other sensing platforms. Another critical limitation of the model is related to the parameterization of *χ*_p_(*π*) for the different particle populations. The lack information on the variability of *χ*_p_(*π*) in the surface ocean represents a fundamental knowledge gap in the oceanographic lidar field that limits our ability to constrain the uncertainties associated with modeling *δ* from water column inherent optical properties or retrieving *b*_bp_ from lidar profiles of *β*_p_(*π*). Future efforts to constrain the variability of *χ*_p_(*π*) in the global ocean, such as those recently published by Hu et al. (2020), should be included in any future efforts to develop oceanographic lidar as a tool for remote sensing of aquatic ecosystems.

Variability in $M_{22}^{p}$(*π*) associated with shifts in the bulk properties of marine particles can also influence retrievals of *b*_bp_ made by using the cross‐polarized channel of the spaceborne lidar CALIOP. For these retrievals, estimates of $M_{22}^{p}$(*π*) are required to convert between measurements of cross‐polarized and particulate backscatter at *π*. This has previously been achieved using an empirical relationship between $M_{22}^{p}$(*π*) and *K*_d_ to either parameterize $M_{22}^{p}$(*π*) from independent measurements of *K*_d_ (Behrenfeld et al. 2013) or to justify the elimination of the *K*_d_ and **M**_22_(*π*) terms from the retrieval (Bisson et al. 2021). These assumptions have produced reasonable retrievals of *b*_bp_, but the mechanistic link between $M_{22}^{p}$(*π*), an intensive property that is independent of particle concentration, and *K*_d_, an extensive property that depends on particle concentration, remains unclear. This makes it difficult to predict when and where assumptions about $M_{22}^{p}$(*π*) may break down and contribute to systematic error in CALIOP *b*_bp_ retrievals. This relationship could potentially result from the broad‐scale covariation between particle concentration, size, and bulk refractive index in the ocean where highly attenuating waters are often associated with suspended mineral sediments and large, bloom‐forming phytoplankton that we showed here to be more depolarizing than small, optically soft species that often predominate in the oligotrophic ocean (Sheldon et al. 1972). Multiple scattering could have also contributed to the relationship between $M_{22}^{p}$(*π*) and *K*_d_, since lidar measurements of *δ* used to derive this relationship were uncorrected for the increase in *δ* with increasing optical depth (Behrenfeld et al. 2013). The broad‐scale agreement found between CALIOP retrievals and in situ measurements of *b*_bp_ suggests that errors associated with multiple scattering either have a negligible influence on CALIOP measurements of *δ* or that the influence of multiple scattering on *δ* is compensated for by error associated with another assumption in the model, for instance the parameterization of *χ*_p_(*π*) (Bisson et al. 2021). The results presented here cannot reject either of these mechanisms; a better understanding of the variability in $M_{22}^{p}$(*π*) and *χ*_p_(*π*) in the global ocean as well as the influence of multiple scattering on in‐water CALIOP measurements is required to better constrain potential sources of error in CALIOP *b*_bp_ retrievals.

The dependence of *δ* on several intensive and extensive particle properties shown here is reminiscent of the chlorophyll retrieval problem for passive ocean color, where spatial variability in the relative contributions of phytoplankton, nonalgal particles, and colored dissolved organic matter to remote sensing reflectance requires regional tuning of ocean color algorithms (Sathyendranath et al. 1989). A conceptually similar approach may be useful for retrieving particle information from lidar measurements of *δ* and for parameterizing *δ* in CALIOP retrievals of *b*_bp_, but this will require additional information to supplement the low degrees‐of‐freedom afforded by lidar measurements at a single wavelength. Just as ocean color algorithm development has relied on extensive field measurements of water column optical and biogeochemical properties to constrain their influence on remote sensing reflectance spectra, polarized lidar retrievals of particle characteristics will require extensive in‐water measurements of **M**_22_(*π*), *β*(*π*), and the particle intensive properties that contribute to their variability. Instruments designed with these measurements in mind are currently unavailable to the ocean science community, representing a major hurdle for the advancement of polarized oceanographic lidar as a routine remote sensing technique.

In conclusion, this study highlights the complexity of the lidar depolarization response to particle intensive and extensive properties. It also highlights the need for other fundamental, parallel measurements when interpreting polarized oceanographic lidar data to constrain the behavior of $M_{22}^{p}$(*π*). Such validation data will provide the degrees of freedom needed to improve lidar‐based retrievals of particle characteristics and particle backscattering in the sea.

## Conflict of Interest

None declared.

## Supporting information


**Fig. S1** Microscope images of particle suspensions used in light scattering experiment. (**a**) 5 *Synechococcus* sp., (**b**) *Thalassiosira weissflogii*, (**c**) diatomaceous earth, (**d**) *Emiliania huxleyi* 6 with attached and detached coccoliths, and **(e)** laboratory calcite. Arrows are used to highlight 7 examples of a rod‐shaped *Synechococcus* sp. cell (green), a calcified *E. huxleyi* cell (yellow), and 8 a free suspended coccolith (blue). Red scale bars are all 25 μm in length

## Data Availability

The data that support the findings of this study are available from the corresponding author upon reasonable request.
