# Supplementary material for: The influence of particle concentration and bulk characteristics on polarized oceanographic lidar measurements
Source: Limnol Oceanogr. 2022 Apr 29;67(6):1374–87. doi: 10.1002/lno.12088 (PMC9545571; doi:10.1002/lno.12088)
Supplement: Supplementary file 1 — Fig. S1 Microscope images of particle suspensions used in light scattering experiment. (a) 5 Synechococcus sp., (b) Thalassiosira weissflogii, (c) diatomaceous earth, (d) Emiliania huxleyi 6 with attached and detached coccoliths, and (e) laboratory calcite. Arrows are used to highlight 7 examples of a rod‐shaped Synechococcus sp. cell (green), a calcified E. huxleyi cell (yellow), and 8 a free suspended coccolith (blue). Red scale bars are all 25 μm in length [file LNO-67-1374-s001.pdf]

Supplementary Material

Supplementary Figures

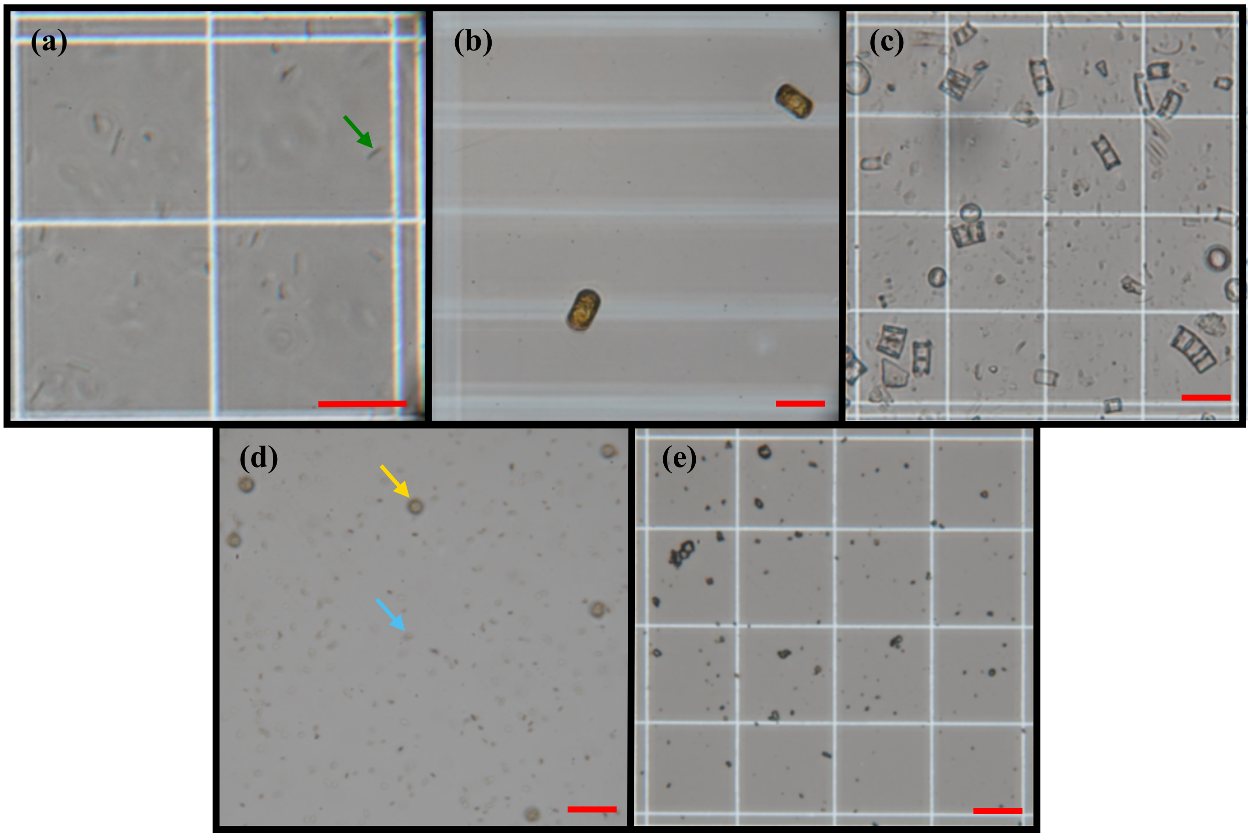

Fig. S1. *Microscope images of particle suspensions used in light scattering experiment.* **(a)** *Synechococcus* sp., **(b)** *Thalassiosira weissflogii*, **(c)** diatomaceous earth, **(d)** *Emiliania huxleyi* with attached and detached coccoliths, and **(e)** laboratory calcite. Arrows are used to highlight examples of a rod-shaped *Synechococcus* sp. cell (green), a calcified *E. huxleyi* cell (yellow), and a free suspended coccolith (blue). Red scale bars are all 25 μm in length
